# Haematologic malignancies with unfavourable gene mutations benefit from donor lymphocyte infusion with/without decitabine for prophylaxis of relapse after allogeneic HSCT: A pilot study

**DOI:** 10.1002/cam4.3763

**Published:** 2021-05-01

**Authors:** Rui Zhang, Lili Wang, Peng Chen, Xiaoning Gao, Shuhong Wang, Fei Li, Liping Dou, Chunji Gao, Yan Li, Daihong Liu

**Affiliations:** ^1^ Department of Hematology Chinese PLA General Hospital Beijing China; ^2^ Department of Hematology People's Hospital of Cangzhou Hebei China; ^3^ Department of Hematology the 7th Medical Center of Chinese PLA General Hospital Beijing China; ^4^ Department of Hematology Peking University Third Hospital Beijing China

**Keywords:** allogeneic peripheral blood stem cell transplantation, decitabine, donor lymphocyte infusion, relapse, unfavourable gene mutations

## Abstract

Relapse is the main cause of treatment failure for leukaemia patients with unfavourable gene mutations who receive allogeneic haematopoietic stem cell transplantation (allo‐HSCT). There is no consensus on the indication of donor lymphocyte infusion (DLI) for prophylaxis of relapse after allo‐HSCT. To evaluate the tolerance and efficacy of prophylactic DLI in patients with unfavourable gene mutations such as FLT3‐ITD, TP53, ASXL1, DNMT3A or TET2, we performed a prospective, single‐arm study. Prophylactic use of decitabine followed by DLI was planned in patients with TP53 or epigenetic modifier gene mutations. The prophylaxis was planned in 46 recipients: it was administered in 28 patients and it was not administered in 18 patients due to contraindications. No DLI‐associated pancytopenia was observed. The cumulative incidences of grade II–IV and III–IV acute graft‐versus‐host disease (GVHD) at 100 days post‐DLI were 25.8% and 11.0%, respectively. The rates of chronic GVHD, non‐relapse mortality and relapse at 3 years post‐DLI were 21.6%, 25.0% and 26.1%, respectively. The 3‐year relapse‐free survival and overall survival (OS) rates were 48.9% and 48.2%, respectively. Acute GVHD (HR: 2.30, *p* = 0.016) and relapse (HR: 2.46, *p* = 0.003) after DLI were independently associated with inferior OS. Data in the current study showed the feasibility of prophylactic DLI with/without decitabine in the early stage after allo‐HSCT in patients with unfavourable gene mutations.

## Graphical abstract

This prospective, single‐arm, pilot study investigated the efficacy and feasibility of prophylactic DLI and showed that prophylactic DLI could effectively prevent relapse without increasing the incidence of GVHD or NRM for patients with unfavourable gene mutations after allo‐HSCT. In addition, prophylactic decitabine followed by DLI showed potential safety and efficacy without impact on hematologic reconstitution and with acceptable incidence of DLI‐related aGVHD.

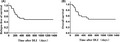

## 1 INTRODUCTION

In the past decade, great progress has been made in the risk stratification of acute leukaemia and myelodysplastic syndrome (MDS) with the development of next‐generation sequencing (NGS) technology.^1^, ^2^, ^3^ Gene mutations, such as FMS‐like tyrosine kinase 3 with internal tandem duplications (FLT3‐ITD), tumour suppressor gene P53 (TP53), additional sex combs‐like 1 (ASXL1), DNA methyltransferase 3A (DNMT3A) and 10–11 translocation‐2 (TET2) have been identified as high‐risk molecular markers for acute leukaemia with a low rate of remission following chemotherapy and short survival.^4^, ^5^, ^6^, ^7^ FLT3‐ITD, TP53 and ASXL1 mutations have been documented as novel molecular risk stratification markers for AML in the National Comprehensive Cancer Network (NCCN) guidelines.^8^ In general, the prognosis of acute myeloid leukaemia (AML) patients with FLT3‐ITD is poor. In patients who achieve complete remission after induction chemotherapy, the rate of relapse is as high as 70%.^9^ The impact of TP53 on the prognosis of acute leukaemia is much worse, and this mutation outweighs all other adverse cytogenetics, such as complex karyotypes and monosomal karyotypes. For patients with TP53, the rates of complete remission are as low as 28% and the rate of long‐term survival is approximately 7%.^10^ The epigenetic modifier gene mutations, ASXL1, DNMT3A and TET2 (ADT) recurrently occur in AML/MDS and result in a dismal prognosis^11^, ^12^ with a low rate of remission and short progression‐free survival.^13^, ^14^, ^15^ Allogeneic haematopoietic stem cell transplantation (allo‐HSCT) remains the only potential cure for patients with unfavourable gene mutations. However, the rate of relapse after allo‐HSCT is up to 50%–60%, and the long‐term relapse‐free survival (RFS) is less than 30%; RFS is as low only 7%–12% for patients with TP53.^10^, ^16^, ^17^

Measures to reduce the relapse rate after allo‐HSCT consist of intervention for patients with positive minimal residual disease (MRD) or prophylactic strategies for those with potential high‐risk characteristics, such as non‐remission status prior to transplantation. Donor lymphocyte infusion (DLI) has been proven to be effective preventing leukaemia relapse after allo‐HSCT with a graft‐versus‐leukaemia (GVL) effect.^18^, ^19^ However, DLI is limited by fatal graft‐versus‐host disease (GVHD), pancytopenia and infections after infusion causing an increase in non‐relapse mortality (NRM). Our previous studies have modified the DLI procedure by using granulocyte colony‐stimulating factor (G‐CSF)‐primed donor peripheral blood stem cells (PBSCs) instead of steady‐state lymphocytes and short‐term immunosuppressants. Improved tolerance and undamaged efficacy were documented in reducing the risk of relapse in patients with very high‐risk leukaemia/lymphoma after transplantation, including both HLA‐matched sibling donor (MSD) and HLA‐haploidentical donor (HID) HSCT.^20^, ^21^ For patients with unfavourable gene mutations lacking targeted therapy, prophylactic DLI in the early stage after allo‐HSCT is a potential feasible strategy to reduce the recurrence of malignancy. Furthermore, the response to hypomethylating agents (HMAs) has been reported in patients with TP53, ASXL1, DNMT3A and TET2.^22^, ^23^ Here, we explore the prophylactic use of decitabine followed by DLI, as D + DLI, for patients with TP53, ASXL1, DNMT3A or TET2 mutations. Data on the tolerance and efficacy of prophylactic DLI in 28 patients are presented. Twelve additional patients were treated contemporaneously under the same transplantation protocol and had been planned for prophylaxis; however, they did not receive prophylaxis due to contraindications before prophylaxis was to be administered. The outcomes of these 12 patients are also analysed to provide a lateral reference for NRM and other toxicities (Supporting Information).

## 2 METHODS

### 2.1 Patients and study design

**FIGURE 1:**
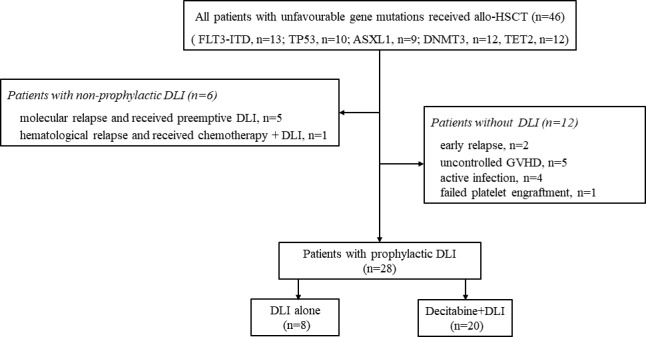
Outline of the study selection. Forty‐six patients were eligible for inclusion. Six patients with molecular or haematological relapse received preemptive DLI or therapeutic DLI and 12 patients did not receive the scheduled prophylactic DLI due to contraindications were excluded. Twenty‐eight patients received prophylactic DLI. Allo‐HSCT, allogeneic haematopoietic stem cell transplantation; DLI, donor lymphocyte infusion; MRD, minimal residual disease; GVHD, graft‐versus‐host disease

This was a prospective, single‐arm study involving a total of 46 patients diagnosed with haematological malignancies with at least one unfavourable molecular mutation (FLT3‐ITD, TP53, ASXL1, DNMT3A or TET2) who received their first allo‐HSCT in our centre between Oct 2014 and Dec 2018 (Figure 1). Prophylactic G‐CSF‐primed DLI after transplantation was planned for all patients at +30~+60 days for MSD‐HSCT and +60~+90 days for HID‐HSCT. Six patients with molecular relapse or haematological relapse before the planned prophylactic DLI who received pre‐emptive or therapeutic DLI were not enrolled, and 12 additional patients did not receive the planned prophylactic DLI due to contraindications (Supporting Information). Prophylactic DLI after transplantation was carried out in 28 patients; 8 patients received DLI and 20 patients received D + DLI (Figure 1). The timing of this prophylaxis was delayed in eight patients due to infection (n = 1) and acute GVHD (n = 7). Fifteen patients have been reported previously^21^ whose outcomes from another 2 years of observation were described in this study. All surviving patients were followed up to 31 May 2020, and there was a median of 486 (156–1759) days of follow‐up after transplantation.

### 2.2 Conditioning regimen and GVHD prophylaxis

Patients without organ dysfunction received a modified Bu/Cy regimen consisting busulphan, carmustine, cytarabine and cyclophosphamide, and patients with organ dysfunction during previous chemotherapy received busulphan and fludarabine in place of cyclophosphamide in the Bu/Cy regimen. For patients with refractory B cell acute lymphoblastic leukaemia, the TBI/Cy regimen was used, which consisted of total body irradiation, cytarabine and Cy, as previously reported.^20^ For recipients of HID‐HSCT, ATG (thymoglobulin, rabbit; Genzyme Europe BV; 2.5 mg/kg/day, days −5 to −2) was used in all recipients. ATG (2.5 mg/kg/day, days −5 to −4) was used in cases where either the donor or the recipient were above 40 years old for recipients of MSD‐HSCT. GVHD prophylaxis using CsA, mycophenolate mofetil, short‐term MTX and supportive care was administered as previously reported.^24^

### 2.3 Prophylactic DLI

Molecular monitoring of the mutations was routinely performed in all patients at +30 days, and they were all negative. Then, patients were routinely monitored for bone marrow morphology, MRD detected by flow cytometry and chimaerism status at +2 m, +3 m, +4.5 m, +6 m, and then, every 6 months. All 28 patients had 100% donor chimaerism before prophylactic DLI. The planned prophylactic DLI was implemented between +30~+60 days after transplantation for MSD‐HSCT recipients and +60~+90 days for HID‐HSCT recipients. If the patient developed acute GVHD before or around the timing of prophylaxis, DLI was delayed to 4 weeks after CR of GVHD. Six patients with a single mutation of FLT3‐ITD who were not tolerant to sorafenib prior to transplantation were scheduled for prophylactic DLI. Patients with any mutations in TP53, ASXL1, DNMT3A or TET2, including co‐occurrence with FLT3‐ITD, were assigned to the D + DLI group: 5 days of decitabine treatment (10 mg/m^2^/d from day 1 to day 5, Chia Tai TianQing Pharmaceutical Co., Ltd) followed by DLI on day 7. The G‐CSF mobilised PBSCs that had been counted and cryopreserved at the time of graft collection were infused at a single target dose of 2 × 10^7^ CD3+ cells/kg recipient.^21^ That was guaranteed by calculating the numbers of cells to be thawed and infused according to the cell count and the real‐time body weight of recipient. All of the patients who received prophylactic DLI before day +60 (MSD‐HSCT) or day +90 (HID‐HSCT), received CsA for routine prevention of acute GVHD with a trough concentration of 150–250 ng/ml.^20^ That concentration was maintained for 4 weeks in MSD‐HSCT cases and 6–8 weeks in HID‐HSCT cases and discontinued within 2 weeks when no DLI‐associated GVHD occurred. For patients who received delayed prophylactic DLI due to contraindications, CsA was administered from day +1 after DLI for prevention of DLI‐associated GVHD if their immunosuppressants were already discontinued. The durations of short‐term CsA were the same as above.

### 2.4 Definitions and endpoints

Acute GVHD and chronic GVHD after HSCT and post‐DLI GVHD were assessed as previously defined.^25^, ^26^, ^27^ Relapse was defined as the haematologic recurrence of leukaemia. NRM was defined as death from any cause without disease relapse. The time points before and after transplantation or DLI are presented by ‘−’ or ‘+’ signs. The cumulative incidences of post‐DLI relapse, acute GVHD and chronic GVHD were the primary endpoints. The secondary endpoints were the cumulative incidence of NRM, OS and RFS.

### 2.5 Statistical analyses

The demographic and clinical characteristics were summarised using descriptive statistics. Clinical features between two groups were compared using the chi‐square test for categorical variables, and Fisher's exact test for an event with the expected frequency of <5 in any 2 × 2 tables cells. Continuous variables were compared with Student's *t* test or non‐parametric Mann–Whitney *U* test. The cumulative incidences of relapse (CIR), GVHD and NRM were calculated using cumulative incidence curves to accommodate competing risks by using Gray's method.^28^, ^29^ The Fine‐Gray hazards model was used for CIR, GVHD and NRM in the univariate and multivariate analyses. Survival analysis was performed by the Kaplan–Meier method with the log‐rank test for univariate analysis and a Cox proportional hazard model was used to assess the prognostic significance of the clinical variables. Variables for univariate analysis of risk for relapse, GVHD, NRM, OS and RFS are shown in Table 2, and all variables associated with *p* < 0.20 by univariate analysis were included in the multivariate analysis. A two‐sided *p* < 0.05 was considered statistically significant. All statistical analyses were performed with R statistical software and the cmprsk package (Comprehensive R Archive Network, TU Wien), Stata 14.0 software (Stata Corporation) and SPSS 20.0 software (IBM Corporation).

## 3 RESULTS

### 3.1 Clinical characteristics of patients and implementation of prophylactic DLI

Twenty‐eight patients with a median age of 36.5 (14–64) years received prophylactic DLI in this study (Figure 1 and Table 1). All patients achieved sustained neutrophil recovery at a median of 12 (9–17) days and platelet engraftment at a median of 14 (9–34) days. Prophylactic DLI was administered at a median time of 73 (35–236) days after transplantation. Twenty (71.4%) patients received prophylactic DLI according to the scheduled timing. Seven patients developed grade I–II acute GVHD at approximately day 27 (+18~+81) and received postponed prophylactic DLI at a median of 116 (96–236) days after transplantation, once the acute GVHD was well controlled. One patient was administered postponed prophylactic DLI at +95 days after MSD‐HSCT due to invasive fungal disease occurring at day +59. The dose of CD3+ cells for infusion was 2 × 10^7^/kg.

**TABLE 1:** Characteristics of prophylactic DLI patients

Characteristics	Prophylactic DLI (n = 28) (n, %)
Patient's age, years, median (range)	36.5 (14, 64)
Patient's age	
<40 years	16 (57.1)
≥40 years	12 (42.9)
Gender	
Male	16 (57.1)
Female	12 (42.9)
Diagnosis	
AML	23 (82.2)
MDS	2 (7.1)
ALL	3 (10.7)
Unfavourable gene mutations	
FLT3‐ITD	8 (28.6)
TP53	7 (25.0)
ASXL1	5 (17.9)
DNMT3A	9 (32.1)
TET2	9 (32.1)
FLT3‐ITD and DNMT3A	4 (14.3)
ASXL1 and DNMT3A	1 (3.6)
ASXL1 and TET2	2 (7.1)
DNMT3A and TET2	1 (3.6)
TP53, ASXL1 and DNMT3A	1 (3.6)
Prophylaxis for relapse	
DLI	8 (28.6%)
DAC + DLI	20 (71.4%)
HSCT type	
MSD	10 (35.7)
HID	18 (64.3)
Disease status at transplantation	
CR1	26 (92.9)
≤2 induction chemotherapy	19
≥3 induction chemotherapy	7
NR	2 (7.1)
Primary induction failure	1
Untreated MDS	1
Interval from diagnosis to transplantation, days, median (range)	147 (84–574)
Conditioning regimen	
Modified Bu/Cy	27 (96.4)
Bu/Fu	0 (0.0)
TBI/Cy	1 (3.6)
Donor's age, median, years (range)	34 (9–61)
Donor's age	
<40 years	18 (64.3)
≥40 years	10 (35.7)
Donor–recipient gender match	
Female to male	3 (10.7)
Female to female	6 (21.4)
Male to female	6 (21.4)
Male to male	13 (46.4)
Graft	
MNCs, median, ×10^8^/kg (range)	8.99 (5.2–19.32)
CD34+, median, ×10^6^/kg (range)	4.42 (2–9.32)

Abbreviations: ALL, acute lymphoblastic leukaemia; AML, acute myeloid leukaemia; Bu, busulphan; CR, complete remission; Cy, cyclophosphamide; DAC, decitabine; DLI, prophylactic donor lymphocyte infusion; Fu, fludarabine; HID, HLA‐haploidentical donor; HLA‐matched sibling donor; HSCT, hematopoietic stem cell transplantation; MDS, myelodysplastic syndrome; MNCs, mononuclear cell counts; MSD; NR, non‐remission; TBI, total body irradiation.

DNMT3A and TET2 were the most common mutations, both with a frequency of 32.1% (9/28), followed by FLT3‐ITD in 28.6% (8/28), TP53 in 25.0% (7/28) and ASXL1 in 17.9 (5/28). DNMT3A mutations usually co‐occurred with other mutations (88.9%, 8/9) (Table 1). A total of 20 patients with any mutations of TP53, ASXL1, DNMT3A or TET2 received D + DLI as planned, while there were three patients, including two patients with TET2 mutations and one patient with FLT3‐ITD and DNMT3A mutations who fulfilled the criteria for D + DLI, but received DLI due to poor performance status; these patients were analysed in the DLI group. Six patients with a single FLT3‐ITD mutation who were not tolerant to sorafenib prior to transplantation were scheduled for DLI. Two of them did not receive DLI prophylaxis due to persistent acute GVHD.

### 3.2 Outcomes of the prophylactic DLI recipients

#### 3.2.1 GVHD

**FIGURE 2:**
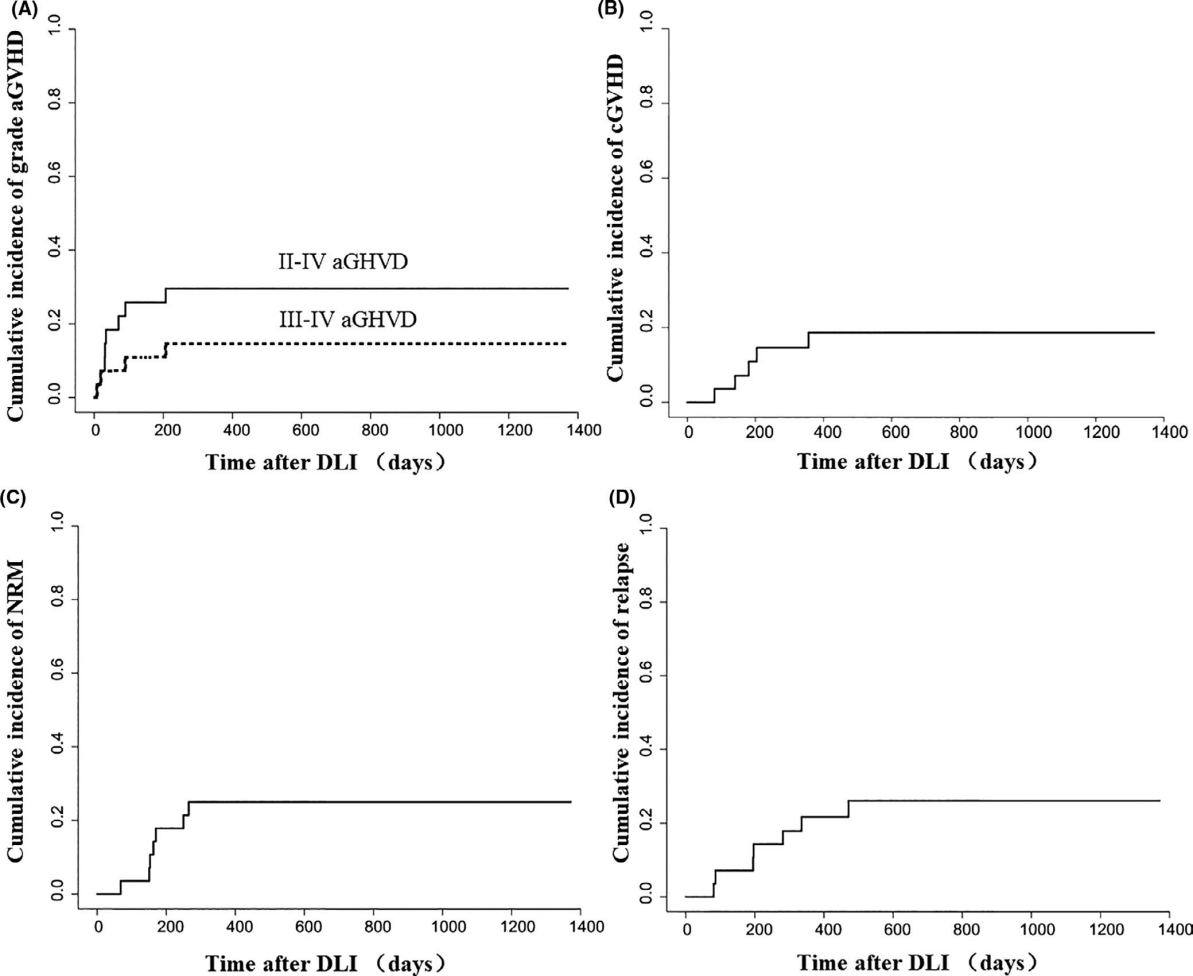
Cumulative incidence of aGVHD (A), cGVHD (B), NRM (C) and relapse (D) of prophylactic DLI recipients. aGVHD, acute graft‐versus‐host disease; cGVHD, chronic graft‐versus‐host disease; DLI, donor lymphocyte infusion; NRM, non‐relapse mortality

No DLI‐associated pancytopenia was documented in the 28 patients. Thirteen patients (46.4%) developed acute GVHD at a median of 31 (5–207) days after prophylactic DLI (grade I in five cases, grade II in four cases, grade III in four cases) and eight (61.5%) of them had previous aGVHD at a median of day +29 (+18~+81) after transplantation and before DLI (grade I in three cases, grade II in four cases, grade III in one case). The cumulative incidences (CIs) of grade II–IV and III–IV acute GVHD at 100 days post‐DLI were 25.8% (95% CI, 24.4%–27.3%) and 11.0% (95% CI, 10.3%–11.7%), respectively (Figure 2A). Chronic GVHD occurred in five (17.9%) recipients at a median of 180 (79–356) days after prophylactic DLI; four patients had mildly involved skin and one had severely involved skin, mouth, eyes and lung. Two patients with chronic GVHD had previous acute GVHD after DLI. The CIs of chronic GVHD at 6‐month, 1‐year and 3‐year post‐DLI were 10.9% (95% CI, 10.2%–11.6%), 18.6% (95% CI, 17.4%–19.8%) and 21.6% (95% CI, 19.8%–23.4%), respectively (Figure 2B). In univariate analyses, there were no factors significantly correlated with the risk of occurrence of grade II–IV acute GVHD, grade III–IV acute GVHD or chronic GVHD after prophylactic DLI (Table 2). There were no independent risk factors for either acute GVHD or chronic GVHD in multivariate analyses (data not shown).

**TABLE 2:** Univariate analysis for the outcomes after prophylactic DLI (n = 28)

Characteristics	Grade 2–4 aGVHD		Grade 3–4 aGVHD		cGVHD		NRM		CIR		RFS		OS	
	HR (95% CI)	*p*	HR (95% CI)	*p*	HR (95% CI)	*p*	HR (95% CI)	*p*	HR (95% CI)	*p*	HR (95% CI)	*p*	HR (95% CI)	*p*
**Age**, ≥40 years vs. <40 years	1.43 (0.37–5.56)	0.604	**4.24 (0.49–37.04)**	**0.191**	0.79 (0.14–4.59)	0.791	**3.32 (0.65–16.97)**	**0.149**	**0.15 (0.02–1.24)**	**0.078**	0. 83 (0.29–2.40)	0.735	0. 80 (0.28–2.32)	0.686
**Gender**, female vs. male	1.43 (0.37–5.60)	0.605	0.43 (0.05–3.80)	0.446	0.80 (0.14–4.54)	0.801	**0.18 (0.02–1.41)**	**0.103**	0.98 (0.22–4.46)	0.982	**0. 39 (0.12–1.27)**	**0.118**	**0. 39 (0.12–1.24)**	**0.110**
**pDLI**, D + DLI vs. DLI	**0.35 (0.09–1.35)**	**0.129**	0.41 (0.06–2.72)	0.357	1.52 (0.17–13.57)	0.710	**0.25 (0.06–1.07)**	**0.061**	2.49 (0.26–23.74)	0.426	0. 52 (0.17–1.56)	0.242	0. 53 (0.18–1.58)	0.255
**CR course**, ≥3 vs. <2	1.95 (0.47–8.06)	0.355	**8.50 (0.86–84.14)**	**0.067**	0.76 (0.08–6.75)	0.802	**2.94 (0.63–13.75)**	**0.171**	1.39 (0.28–7.01)	0.687	**2.28 (0.71–7.31)**	**0.165**	**2.17 (0.68–6.90)**	**0.189**
**FLT3‐ITD^mut^**, yes vs. no	**2.68 (0.69–10.37)**	**0.153**	2.33 (0.34–16.15)	0.391	**3.83 (0.64–22.79)**	**0.141**	0.78 (0.16–3.81)	0.765	0.77 (0.16–3.81)	0.748	0. 70 (0.22–2.23)	0.545	0. 65 (0.20–2.09)	0.472
**TP53^mut^**, yes vs. no	0.34 (0.05–2.51)	0.292	0.94 (0.11–8.36)	0.954	0.76 (0.08–7.56)	0.814	0.49 (0.05–4.47)	0.531	1.28 (0.26–6.25)	0.756	0. 82 (0.23–2.94)	0.759	0. 82 (0.23–2.94)	0.760
**ASXL1^mut^**, yes vs. no	0	‐	0	‐	1.00 (0.14–7.26)	0.998	0.66 (0.10–4.52)	0.673	2.04 (0.42–9.95)	0.378	1.22 (0.34–4.36)	0.765	1.29 (0.36–4.65)	0.697
**DNMT3A^mut^**, yes vs. no	0.31 (0.04–2.65)	0.282	0	‐	1.43 (0.25–8.16)	0.685	0.30 (0.04–2.31)	0.248	0.90 (0.18–4.52)	0.894	0. 48 (0.13–1.71)	0.256	0. 49 (0.14–1.75)	0.269
**TET2^mut^**, yes vs. no	1.21 (0.30–4.86)	0.789	0	‐	0.47 (0.06–3.75)	0.473	1.64 (0.38–7.00)	0.507	0.71 (0.16–3.24)	0.659	1.05 (0.35–3.15)	0.925	1.06 (0.35–3.16)	0.921
**HSCT type**, HID vs. MSD	**4.50 (0.63–32.26)**	**0.135**	1.68 (1.19–14.80)	0.638	0.36 (0.06–2.09)	0.255	‐	‐	0.77 (0.18–3.37)	0.734	**2.95 (0.82–10.66)**	**0.098**	**3.18 (0.87–11.60)**	**0.079**
**Disease status at HSCT**, NR vs. CR	2.16 (0.28–16.95)	0.463	0	‐	0	‐	2.30 (0.36–14.61)	0.377	2.30 (0.36–14.61)	0.377	**2.71 (0.59–12.45)**	**0.199**	2.70 (0.59–12.38)	0.202
**aGVHD before DLI**, yes vs. no	2.42 (0.51–11.52)	0.264	2.33 (0.26–20.69)	0.446	**0.16 (0.02–1.37)**	**0.096**	**5.49 (0.71–42.67)**	**0.103**	0.93 (0.21–4.11)	0.921	**2.27 (0.71–7.26)**	**0.166**	**2.24 (0.70–7.17)**	**0.172**
**Donor age**, ≥40 years vs. <40 years	**0.21 (0.03–1.75)**	**0.149**	0	‐	1.07 (0.20–5.83)	0.937	**0.25 (0.03–1.83)**	**0.171**	2.36 (0.55–10.07)	0.245	0. 76 (0.25–2.28)	0.626	0. 74 (0.25–2.22)	0.588
**aGVHD after DLI**, yes vs. no	**‐**	**‐**	**‐**	**‐**	**‐**	**‐**	**‐**	**‐**	**‐**	**‐**	**2.13 (0.73–6.19)**	**0.167**	**2.18 (0.75–6.38)**	**0.153**
**cGVHD after DLI**, yes vs. no	**‐**	**‐**	**‐**	**‐**	**‐**	**‐**	**‐**	**‐**	**‐**	**‐**	1.24 (0.34–4.45)	0.744	1.23 (0.34–4.42)	0.749
**Relapse after DLI**, yes vs. no	**‐**	**‐**	**‐**	**‐**	**‐**	**‐**	**‐**	**‐**	**‐**	**‐**	**‐**	**‐**	**4.15 (1.44–11.99)**	**0.009**

Factors marked in bold were included into multivariate analyses (*p* < 0.20). A two‐sided *p* < 0.05 was considered statistically significant.

Abbreviations: aGVHD, acute graft‐versus‐host disease; cGVHD, chronic graft‐versus‐host disease; CI, confidence interval; CIR, cumulative incidence of relapse; CR, complete remission; D + DLI, decitabine and prophylactic donor lymphocyte infusion; HID, HLA‐haploidentical donor; HR, hazard ratio; HSCT, hematopoietic stem cell transplantation; MSD, HLA‐matched sibling donor; NR, non‐remission; NRM, non‐relapse mortality; OS, overall survival; pDLI, prophylactic donor lymphocyte infusion; RFS, relapse‐free survival.

#### 3.2.2 NRM

A total of seven patients (7/28, 25.0%) died of non‐relapse complications. Three of them died of acute GVHD (one patient refused to receive medical treatment), two died of infectious pneumonia, one died of capillary leak syndrome and one died of thrombotic microangiopathy (TMA). The 6‐month, 1‐year and 3‐year CIs of NRM were 17.9% (95% CI, 16.8%–18.9%), 25.0% (95% CI, 23.6%–26.4%) and 25.0% (95% CI, 23.6%–26.4%), respectively (Figure 2C). No independent risk factors were found in multivariate analyses (data not shown). A trend in reduced NRM was observed in the D + DLI group compared with the DLI alone group in univariate analyses, though the difference was not significant (HR: 0.25, 95% CI: 0.06–1.07, *p* = 0.061) (Table 2).

#### 3.2.3 Recurrence of haematologic malignancy

Seven patients (25.0%) relapsed at a median of 196 (81–471) days after prophylactic DLI and 264 (133–542) days after transplantation. Of these seven patients, one with the TET2 mutation was in NR status prior to transplantation, and another six patients were in CR1 status (one with FLT‐ITD and DNMT3A mutations, one with ASXL1 and TET2 mutations, one with the ASXL1 mutation, one with the DNMT3A mutation and two with the TP53 mutation). All seven patients died of relapse at a median of 246 (103–547) days after prophylactic DLI and 315 (165–649) days after transplantation. The 6‐month, 1‐year and 3‐year CIs of relapse after prophylactic DLI were 7.1% (95% CI, 6.7%–7.6%), 21.7% (95% CI, 20.4%–23.0%) and 26.1% (95% CI, 24.6%–27.6%), respectively (Figure 2D). In univariate and multivariate analyses, no risk factors including the types and co‐occurrence of unfavourable gene mutations were found to be significantly correlated with relapse (Table 2).

#### 3.2.4 Survival

**FIGURE 3:**
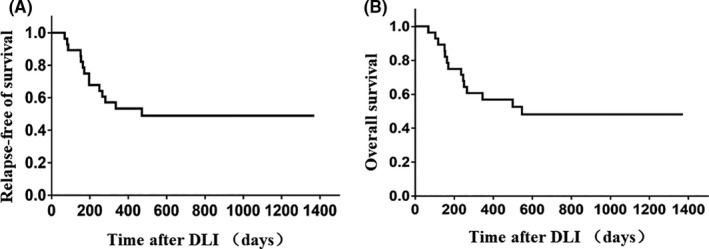
Survival of prophylactic DLI recipients (A & B). DLI, donor lymphocyte infusion; OS, overall survival; RFS, relapse‐free survival

At the time of analysis, 14 (50%) of the 28 prophylactic DLI recipients were alive and free of relapse and GVHD at a median of 940 (294–1371) days after DLI and 1037 (436–1467) days after transplantation. The estimated median RFS and OS times after DLI were 471 and 547 days, respectively. In Kaplan–Meier estimates, the 6‐month, 1‐year and 3‐year RFS rates were 75.0% (95% CI, 54.6%–87.2%), 53.3% (95% CI, 33.5%–69.7%) and 48.9% (95% CI, 29.2%–66.0%), and for OS were 75.0% (95% CI, 54.6%–87.2%), 56.9% (95% CI, 36.8%–72.8%) and 48.2% (95% CI, 28.4%–65.5%), respectively (Figure 3A,B). Univariate analyses demonstrated that relapse after DLI was significantly correlated with unfavourable OS (HR: 4.15, 95% CI: 1.44–11.99, *p* = 0.009) (Table 2). In multivariate analyses, acute GVHD and relapse after DLI (HR: 2.30, 95% CI: 1.17–4.52, *p* = 0.016; HR: 2.46, 95% CI, 1.36–4.46, *p* = 0.003, respectively) were independently associated with poor OS. None of the risk factors were tested significantly correlated with RFS (data not shown).

#### 3.2.5 Comparison of the outcomes between the D + DLI and DLI group

No significant differences in the baseline characteristics (Table 1) between the D + DLI (n = 20) and DLI (n = 8) groups were found (data not shown). Univariate analysis of GVHD, CIR, NRM, RFS and OS demonstrated no notable differences between these two groups (*p* > 0.05, Table 2). In addition, there were three HID‐HSCT recipients (two patients with TET2 mutations, Patient 1 and Patient 2; one patient with FLT3‐ITD and DNMT3A mutations, Patient 3) who fulfilled the criteria for D + DLI but who received DLI due to poor performance status. Patient 2 developed grade II acute GVHD involving the intestinal and liver 34 days after DLI. Patient 3, who was without previous acute GVHD, developed grade II acute GVHD involving the skin and upper gastrointestinal tract at 30 days after DLI. All of these three patients died of non‐relapse reasons. Patient 1 died of interstitial pneumonia at 170 days after DLI. Patient 2 and Patient 3 died of DLI‐related GVHD at 68 days and 250 days after DLI, respectively. Of the 20 recipients of D + DLI, 55.0% (11/20) had acute GVHD before DLI, and 45.0% (9/20) developed acute GVHD after DLI, including six patients (6/11, 54.5%) who had previous acute GVHD. At the end of follow‐up, 11 of these 20 patients (55.0%) were alive and free of GVHD; they relapsed at a median of 880 (294–1289) days post‐DLI. Six patients died of relapse and one died of TMA, while only two patients died of DLI‐related GVHD at 151 days and 265 days after DLI.

## 4 DISCUSSION

Relapse remains the primary cause of treatment failure and mortality after allo‐HSCT for patients with unfavourable gene mutations. In the context of the lack of targeted therapy, this prospective, single‐arm, pilot study investigated the feasibility and efficacy of prophylactic DLI with/without decitabine in the early stage of transplantation. It showed that prophylactic DLI could effectively prevent relapse without increasing the incidence of GVHD or NRM in these specific high‐risk patients. In patients who received prophylactic decitabine followed by DLI, the tolerance in early haematopoietic reconstitution after transplantation was also demonstrated.

Prophylactic DLI has been shown to be effective in preventing relapse in high‐risk recipients of allo‐HSCT.^30^, ^31^ However, given the potentially fatal complications associated with DLI, the selection of candidates for prophylactic DLI has been careful with balancing the risks of recurrence and complications.^32^ For patients with refractory/relapsed acute leukaemia, the CIR after allo‐HSCT is above 50% and the RFS is only 25%–30%.^33^, ^34^ These patients have strong indications for prophylactic DLI.^31^ In previous studies, we established a modified prophylactic DLI setting with G‐CSF‐primed progenitors instead of steady‐state lymphocytes and short‐term immunosuppression after DLI.^20^, ^21^ The feasibility of prophylactic DLI in the haplo‐HSCT setting and the anti‐leukaemic efficacy in refractory/relapsed acute leukaemia have been proven.^20^ Unfavourable gene mutations predict a high incidence of relapse after transplantation for patients either in CR or with refractory/relapsed status prior to transplantation.^10^, ^35^, ^36^, ^37^ However, the tolerance and efficacy of prophylactic D + DLI in these patients in the early stage after transplantation is unclear. Although isolated DNMT3A or TET2 mutations was not included in high‐risk molecular stratifications like ASXL1,^8^ patients with any epigenetic mutations of ASXL1, DNMT3A or TET2 (ADT) had inferior overall survival compared with patients without ADT mutations,^38^, ^39^, ^40^, ^41^ regardless of whether they received HSCT. In the current study, the 3‐year RFS of patients with ADT mutations receiving prophylactic DLI was 46.2%. TP53 mutation was an independent poor prognostic factor and the 3‐year RFS of MDS/AML patients with TP53 mutations was as low as 0%–7% even if they received allo‐HSCT.^10^, ^17^, ^42^ In the current study, the 3‐year RFS of seven patients with TP53 mutations with the above prophylaxis was 38.1%. Although relapse and survival could not be directly compared with other reports, the results of the current study indicated a promising outcome in patients with unfavourable gene mutations.

For AML patients with FLT3‐ITD mutations, the long‐term RFS was only 20%–30% and the median survival was 8.6 months when treated with cytotoxic chemotherapy.^43^ Despite recent advances in FLT3 inhibitors, these patients still presented with high rates of early relapse. Their prognosis after allo‐HSCT was dismal, with a 1‐year OS of less than 20%.^35^ Sorafenib is the only targeted FLT3 inhibitor available on the domestic market and its therapeutic effect is not satisfactory.^44^ In the current study, patients with FLT3‐ITD mutations were intolerant to sorafenib prior to transplantation. Therefore, they were scheduled for prophylactic DLI and showed a 3‐year RFS of 62.5%.

In this study, the incidences of DLI‐associated grade II–IV acute GVHD, grade III–IV acute GVHD and chronic GVHD were 25.8%, 11.0% and 21.6%, respectively. In our previous study,^20^ the incidences of DLI‐associated grade II–IV acute GVHD, grade III–IV acute GVHD and chronic GVHD were 55.3%, 10.2% and 52.0%, respectively, in patients with very high‐risk leukaemia/lymphoma after HID‐HSCT. The data from another study in our centre were 45.2% for grade II–IV acute GVHD, 12.6% for grade III–IV acute GVHD and 21.3% for chronic GVHD in patients with very high‐risk AML.^21^ In the patients who received allo‐HSCT without prophylactic DLI in our centre before January 2015,^24^ the incidences of grade II–IV acute GVHD, grade III–IV acute GVHD and chronic GVHD were 35.1%, 14.5% and 38.4%, respectively. Therefore, the incidences of GVHD after prophylactic DLI in this study were comparable to those previously reported. That is, prophylactic DLI did not result in intolerable toxicity in terms of GVHD. The incidence of NRM in this study was 25.0%, which was also comparable to those in our previous two prophylactic DLI studies (24.0% and 27.9%),^20^, ^21^ and to that reported before 2015 (24.0%).^24^ This indicated the tolerability of prophylactic DLI for patients with unfavourable gene mutations in terms of GVHD occurrence and severity.

The combination of decitabine and DLI was shown to be effective as a salvage therapy for AML or MDS that relapses after allo‐HSCT.^45^ However, the safety and efficiency of integrating decitabine into prophylactic D + DLI for high‐risk patients in the early stage of transplantation remain unknown. In the current study, no D + DLI‐associated pancytopenia was documented in a total of 20 patients including 12 patients after HID‐HSCT. In addition, patients in the D + DLI group had a relatively lower incidence of grade II–IV acute GVHD, grade III–IV acute GVHD and severe acute GVHD‐associated NRM (20.0% vs. 50.0%, 10.0% vs. 25.0% and 22.0% vs. 40.0%, respectively). Three patients who fulfilled the criteria for D + DLI received DLI without decitabine due to delayed haematologic reconstitution and poor performance status. The reason for selecting this option was our concern about the haematological toxicity of decitabine that might result in cytopenia and infection‐associated mortality. In fact, on the one hand, the patients in the D + DLI group were safe in terms of haematopoietic recovery with a lower incidence of acute GVHD and NRM. On the other hand, two of the above three patients died of DLI‐associated acute GVHD. Therefore, we speculated that the addition of decitabine to prophylactic DLI could be the reason for the lower incidence of DLI‐associated GVHD in the current study than that in our previous studies. However, this could not be confirmed due to the limited number of cases in the current study. Recent studies have demonstrated that inclusion of decitabine in the conditioning regimen for allo‐HSCT in intermediate‐ and high‐risk MDS/AML patients may lower the incidence of acute GVHD.^46^, ^47^ The underlying mechanism may be associated with the induction regulatory T cells (Tregs) by decitabine.^48^ Therefore, we have reason to infer that the benefits in the D + DLI group were derived from decitabine with the inhibition of adverse gene mutations and immunoregulation of Tregs. To obtain a more definitive conclusion, the role of decitabine in prophylactic DLI needs to be further investigated in an expanded and prospective randomised controlled trial. A recent review showed that HMAs in combination with FLT3 inhibitors increased the ORR of AML patients with FLT3‐ITD mutations.^49^ Concomitant prophylactic use of decitabine or FLT3 inhibitors followed by DLI could be an option with potentially promising efficacy in reducing relapse in these patients after transplant. In addition, previous studies have shown that using single azacitidine or decitabine as maintenance therapy helped prevent relapse in AML/MDS patients post‐HSCT from matched related donors and matched unrelated donors,^50^, ^51^, ^52^ and a new study suggested that rhG‐CSF combined with minimal‐dose decitabine maintenance after allo‐HSCT can reduce the incidence of relapse.^53^

The major limitations of the current study are the limited numbers of patients, and the lack of a control group. Even though all patients with unfavourable gene mutations were scheduled to receive the prophylactic strategy, not all of the patients had the opportunity to receive prophylactic DLI because of early relapse, or persistent GVHD, Nevertheless, the patients enrolled in this study were consecutive with a consistent protocol for prophylactic DLI, which guaranteed the objectivity of the conclusion. In addition, the data of 12 patients who were treated contemporaneously under the same protocol but did not receive DLI due to various contraindications are also shown in Supporting Information. To some extent, these data may provide a reference for GVHD, NRM and other transplantation toxicities. The results of the current study provide us the following insights. First, the strategy to prevent early relapse after transplantation should be intensified prior to transplantation. Second, persistent GVHD and poor haematopoietic reconstitution might not be contraindications for prophylactic D + DLI.

In summary, this study further confirmed the scientific rationality of the modified prophylactic DLI system in our centre. The data demonstrated the safety and feasibility of prophylactic DLI with/without decitabine in patients with unfavourable gene mutations in allo‐HSCT settings with PBSCs as grafts.

## CONFLICT OF INTEREST

The authors declare no potential conflict of interest.

## ETHICS STATEMENT

This study was approved by the Ethics Committee of Chinese PLA General Hospital, and signed informed consents were obtained from all patients prior to transplantation in accordance with principles of Declaration of Helsinki.

## Supporting information

Supplementary Material

## Data Availability

The data that support the findings of this study are available from the corresponding author upon reasonable request.
