# Supplementary material for: Haematologic malignancies with unfavourable gene mutations benefit from donor lymphocyte infusion with/without decitabine for prophylaxis of relapse after allogeneic HSCT: A pilot study
Source: Cancer Med. 2021 May 1;10(10):3165–76. doi: 10.1002/cam4.3763 (PMC8124122; doi:10.1002/cam4.3763)
Supplement: Supplementary file 1 — Supplementary Material [file CAM4-10-3165-s001.docx]

**Supporting Information**

This information has been provided by the authors to give readers additional information about their work.

**Outcomes of the non-DLI patients**

A total of 12 patients with unfavourable gene mutations did not receive planned prophylactic DLI because of early relapse (n = 2), uncontrolled or intermittent GVHD (n = 5), uncontrolled infection (n = 4), and failed platelet engraftment (n = 1). The CIs of II–IV acute GVHD, III–IV acute GVHD, chronic GVHD, NRM, relapse, RFS and OS were noncontrolled compared with those of patients in the prophylactic DLI group (Supporting Table 1). The incidence of grade II–IV acute GVHD in the DLI group was lower than that in the non-DLI group (HR: 0.33, 95% CI: 0.12–0.88, *p* = 0.027), although persistent GVHD was regarded as a contraindication. There were no statistically significant differences in the majority of baseline clinical characteristics between the DLI group and non-DLI group except there were more patients above 40 years old in the non-DLI group (83.3% vs*.* 42.9%, p = 0.018) (Table 1). Hence, age-adjusted analyses were performed, and no significant difference in NRM between the two groups (Supporting Table 1) was found.

| Supporting Table 1. Outcomes of the prophylactic DLI and non–prophylactic DLI recipients | | | | | | | | | | |
| --- | --- | --- | --- | --- | --- | --- | --- | --- | --- | --- |
|  | Prophylactic DLI (n = 28) | | | | Non-prophylactic DLI (n =12) | | | | |  |
|  | day + 100 | 6-month | 1-year | 3-year | day + 100 | 6-month | 1-year | 3-year | univariate | Age adjusted |
|  | %, (95%CI) | %, (95%CI) | %, (95%CI) | %, (95%CI) | %, (95%CI) | %, (95%CI) | %, (95%CI) | %, (95%CI) | HR (95%CI), P | HR (95%CI), P |
| Cumulative Incidences |  |  |  |  |  |  |  |  |  |  |
| 2-4 acute GVHD ^†^ | 25.8 | - | - | - | 66.7 | - | - | - | **0.33 (0.12-0.88), 0.027** | 0.30 (0.08-1.08), 0.066 |
|  | (24.4-27.3) |  |  |  | (22.4-71.0) |  |  |  |  |  |
| 3-4 acute GVHD ^†^ | 11.0 | - | - | - | 14.2 | - | - | - | 0.63 (0.11-3.57), 0.605 | 1.12 (0.19-6.54), 0.899 |
|  | (10.3-11.7) |  |  |  | (10.2-18.4) |  |  |  |  |  |
| chronic GVHD ^†^ | - | 10.9 | 18.6 | 21.6 | - | 8.3 | 16.7 | 25.0 | 0.77 (0.20-3.02), 0.706 | 0.82 (0.21-3.14), 0.771 |
|  |  | (10.2-11.6) | (17.4-19.8) | (19.8-23.4) |  | (6.9-9.7) | (14.2-19.2) | (21,7%-28.3) |  |  |
| NRM | - | 0.0 | 25.0 | 25.0 | - | 16.7 | 33.3 | 33.3 | 0.67 (0.19-2.30), 0.519 | 0.97 (0.28-,31), 0.958 |
|  |  | - | (23.6-26.4) | (23.6-26.4) |  | (14.2-19.2) | (29.2-37.5) | (29.2-37.5) |  |  |
| Relapse | - | 7.1 | 17.9 | 25.8 | - | 33.3 | 33.3 | 33.3 | 0.60 (0.17-2.17), 0.437 | **0.08 (0.01-0.51), 0.008** |
|  |  | (66-7.6) | (16.8-19.0) | (24.3-27.3) |  | (29.2–37.5) | (29.2-37.5) | (29.2-37.5) |  |  |
| RFS | - | 92.9 | 57.1 | 49.2 | - | 50.0 | 33.3 | 33.3 | **0.29 (0.12-0.69), 0.017** | **0.56 (0.32-0.98), 0.041** |
|  |  | (74.3-98.2) | (37.1-72.9) | (29.1-66.7) |  | (20.8-73.6) | (10.3-58.8) | (10.3-58.8) |  |  |
| OS | - | 96.4 | 60.7 | 48.6 | - | 66.7 | 41.7 | 33.3 | 0.49 (0.18-1.31), 0.067 | 0.63 (0.37-1.06), 0.081 |
|  |  | (77.2-99.5) | (40.4-76.0) | (28.9-65.8) |  | (33.7-86.0) | (15.3-66.5) | (10.3-58.8) |  |  |

^†^ for patients in prophylactic DLI evaluated after DLI. DLI, donor lymphocyte infusion; CI, confidence interval; GVHD, acute graft-versus-host disease; NRM, non-relapse mortality; RFS, relapse-free survival; OS, overall survival.
